# Bariatric Surgery Is Protective Against Renal Function Decline in Severely Obese Patients in the Long-Term

**DOI:** 10.1007/s11695-020-05096-w

**Published:** 2020-11-07

**Authors:** Anne Lautenbach, Jan-Wilhelm Wienecke, Fabian Stoll, Nina Riedel, Oliver Mann, Tobias B. Huber, Philipp Busch, Jens Aberle

**Affiliations:** 1 III. Department of Medicine, University Medical Center Hamburg-Eppendorf, Martinistr. 52, 20246 Hamburg, Germany; 2 Department of Psychiatry, Asklepios Clinic North-Ochsenzoll, Langenhorner Chaussee 560, 22419 Hamburg, Germany; 3 Faculty of Life Sciences, Department of Nutrition and Home Economics, Hamburg University of Applied Sciences, Ulmenliet 20, 21033 Hamburg, Germany; 4 Department of General, Visceral and Thoracic Surgery, University Medical Center Hamburg-Eppendorf, Martinistr. 52, 20246 Hamburg, Germany

**Keywords:** Bariatric surgery, Glomerular filtration rate, Renal function, Obesity, Long-term

## Abstract

**Purpose:**

This study aims to assess the long-term renal effects of bariatric surgery (BS) in severely obese patients over a follow-up period of up to 11 years.

**Materials and Methods:**

In a retrospective cohort study including 102 patients, patients were stratified by eGFR at baseline and divided into three groups: (1) reduced, (2) normal, and (3) increased filtration rate. Adjustments for age- and sex-related decline in eGFR were performed. We used uni- and multivariate regression analysis to identify variables that were thought to determine change in eGFR.

**Results:**

Over a median follow-up of 8.5 years (interquartile range 2.7), eGFR declined from 96.1 ± 20.7 to 84.9 ± 21.0 ml/min (*p < 0.001*). Among patients with (1), eGFR remained stable (69.1 ± 19.3 ml/min). Among patients with (2), eGFR declined from 99.7 ± 13.3 ml/min to 88.7 ± 19.4 ml/min (*p < 0.001*). Among patients with (3), eGFR decreased to normal levels (94.2 ± 17.7 ml/min, *p < 0.001*). Age- and sex-adjusted eGFR increased (6.4 ± 14.4 ml/min; *p < 0.05)* among patients with reduced filtration rate. Among patients with normal filtration rate, adjusted eGFR remained stable during follow-up (−1.3 ± 15.2 ml/min; *n.s*.*).* Among patients with increased filtration rate, adjusted eGFR decreased and remained within the normal range (−13.2 ± 12.2 ml/min; *p < 0.001*). Change in eGFR showed a negative correlation with eGFR at baseline (B = −0.31; *p < 0.001*), change in LDL-cholesterol (B = -0.09; *p < 0.05*), and a negative correlation with treatment requiring hypertension (B = -9.36; *p = 0.001*).

**Conclusion:**

BS is protective against renal function decline in severely obese patients in the long term.

## Introduction

Patients with obesity are more likely to develop chronic kidney disease (CKD) and end-stage renal failure [1]. Obesity-induced intrarenal hemodynamic changes include increased renal plasma flow and glomerular filtration rate (GFR) [2, 3]. Compensatory glomerular hyperfiltration is considered an early sign of glomerular disease in obesity and thus plays a central role in obesity-induced CKD [4, 5]. In the long-term though, individuals with a body mass index (BMI) greater than 30 kg/m^2^ have a significant higher risk of glomerular filtration rate decline [6]. The most prevalent obesity-related comorbidities promoting renal injury are systemic hypertension and Type 2 diabetes (T2D) which can lead to diabetic nephropathy and hypertensive nephrosclerosis [1, 7]. Most patients show slow progression of non-nephrotic proteinuria and worsening of renal function, but 10–33% of patients develop end-stage renal failure in the long-term [3]. At-risk patients for a more severe clinical course are those with greater proteinuria, older age and renal dysfunction at onset of obesity-related glomerulopathy (ORG) [2].

The most studied interventions in ORG are renin-angiotensin-aldosterone-system blockade and weight loss [3]. Data from randomized controlled trials demonstrate that many of the clinical and histopathologic alterations can be attenuated with reductions in body fat [1, 3, 8]. These improvements were more pronounced in bariatric surgery-treated patients than in those with weight loss induced by lifestyle methods [9].

While evidence of improved renal function following bariatric surgery (BS) in the short-and mid-term exists [7, 10–14], only very few studies report long-term renal outcomes (> 5 years) for Roux-en-Y gastric bypass (RYGB) and sleeve gastrectomy (SG) [9, 15].

Therefore, in the present study, we addressed the following questions: (1) Does BS improve renal function in the long-term (up to 11 years) in patients with class III obesity and (2) what is the impact of variables such as preoperative age, BMI, HbA1c, eGFR, LDL-cholesterol and obesity-related comorbidities (hypertension, T2D)?

## Materials and Methods

### Study Design

Postoperative follow-up data were retrospectively collected from 330 patients. The median follow-up time was 8.5 years. To provide reasonable comparability between the cases, the available data were allocated 3 “visits” by time in relation to the bariatric procedure. In addition to baseline data 8.3 ± 0.4 (mean ± SD) months before surgery, data from visit 1 were analyzed at 9.8 ± 0.3 (mean ± SD) months (minimum 2.4 months, maximum 19.2 months) and data from visit 2 at 8.2 ± 1.6 (mean ± SD) years (minimum 5 years, maximum 11 years) post BS. If a patient attended the clinic more than once during a time slot defined by this manner, the visit closest to 12-month follow-up was chosen for visit 1.

### Study Population

Male or female patients ≥ 18 years who underwent either SG or RYGB according to the S3 Leitlinie (Guidelines) *Chirurgie der Adipositas* [16] were included in the analysis. Patients with second step procedures during follow-up were considered as having RYGB (*n* = 2). Patients attended our interdisciplinary obesity outpatient clinic between 2009 and 2020. Exclusion criteria included incomplete records, history of specific nephropathies not related to obesity, history of obstructive urinary lithiasis and previous renal surgery. Of an initial population of 330 patients, 228 were excluded (*n* = 102 patients).

### Variables

Data on height, weight, BMI, gender, age, HbA1c, serum albumin, LDL-cholesterol, and renal function parameters were analyzed at baseline and during follow-up visits. Additionally, the prevalence of hypertension and T2D was assessed. T2D was assumed if the diagnosis was pre-existing or if patients required antidiabetic medication or if HbA1c was above 6.5%. Arterial hypertension was assumed if patients required antihypertensive medication. eGFR was calculated using the CKD-EPI formula based on serum creatinine according to Levey et al. [17].

We computed the change in eGFR as the change in eGFR between baseline and each follow-up visit. Albuminuria was defined as urinary albumin excretion above 20 mg/l in spot urine.

### Subgroup Classification

Patients were stratified by eGFR at baseline and divided into three groups based on the values for normal GFR in an age- and gender-matched control group using inulin clearance [18]: (1) reduced filtration rate (GFR below the 25th percentile) (2) normal filtration rate (GFR between the 25th and 75th percentiles) and (3) increased filtration rate (GFR above the 75th percentile).

### Statistical Analysis

Dependent continuous variables were tested using *t* test, dependent categorical variables were tested using McNemar test, and independent categorical variables were tested using chi-squared test. Normality of scale variables was assessed using Shapiro-Wilk test, and homogeneity of variances was tested using Levene’s test for equal variances. Comparisons of continuous or ordinal measurements in case of non-normal distributions were performed using the Mann-Whitney U-test for independent samples and paired sample sign test for paired samples. Comparison of proportions was performed using the chi-squared test. Univariate regression analysis was performed to identify independent variables that determined the change in eGFR at long-time follow-up. Variables found to be statistically significant (*p* < 0.1) in the univariate analysis were included in the multiple linear regression. The values are expressed as mean ± SD. For all statistical tests, a *p < 0.05* was considered statistically significant. Data collection was performed on Microsoft Excel 2007 and 2016. All analyses were conducted using SPSS software, version 23 for Windows.

## Results

### Demographic Data

Baseline characteristics are presented in Table 1. Mean age was 43.6 ± 10.8 years, and 79.4% of patients were female. At visit 1, there were significant reductions in weight (151.6 ± 36.2 versus 109.7 ± 29.0 kg; *p < 0.001*) and BMI (51.3 ± 9.5 versus 37.1 ± 8.0 kg/m^2^; *p < 0.001*), which were maintained at long-term follow-up (111.6 ± 27.8 kg, *p < 0.001* and 37.9 ± 8.5 kg/m^2^, *p < 0.001*) compared with baseline. Mean HbA1c decreased from 6.1 ± 1.0% to 5.6 ± 0.7% at visit 1 and to 5.7 ± 0.9% at visit 2 (*p < 0.001)*. Accordingly, the percentage of patients with T2D decreased from 34.3%\ to 18.6% at visit 1 (*p* < 0.001) and 16.7% at visit 2 (*p* < 0.001). At baseline, 67.0% of patients were treated with at least one antihypertensive drug. At visit 1 and visit 2, 40.0% and 41.0% suffered from treatment requiring hypertension (*p < 0.001* for both visits), respectively. Mean LDL-cholesterol declined from 117.9 ± 31.1 to 108.5 ± 29.4 mg/dl at visit 1 (*p < 0.001*) and 99.8 ± 30.8 mg/dl at visit 2 (*p < 0.001*)*.*

**Table 1:** Anthropometric and biochemical characteristics at baseline and follow-up visits

Parameter	Baseline	Visit 1	*p* value	Visit 2	*p* value
*N*	102	102		102	
Age (year)	43.6 ± 10.8	45.1 ± 10.8		52.5 ± 11.1	
Gender (F, n%)	79	79		79	
Weight (kg)	151.6 ± 36.2	109.7 ± 29.0	< 0.001	111.6 ± 27.8	< 0.001
BMI (kg/m^2^)	51.3 ± 9.5	37.1 ± 8.0	< 0.001	37.9 ± 8.5	< 0.001
HbA1c (%)	6.1 ± 1.0	5.6 ± 0.7	< 0.001	5.7 ± 0.9	< 0.001
T2DM (%)	34.3	18.6	< 0.001	16.7	< 0.001
Hypertension (%)	67.0	40.0	< 0.001	41.0	< 0.001
LDL-Cholesterol (mg/dl)	117.9 ± 31.1	108.5 ± 29.4	< 0.001	99.8 ± 30.8	< 0.001
S-Albumin (mg/dl)	43.4 ± 2.6	39.5 ± 3.3	< 0.05	37.0 ± 2.6	< 0.05
S-Creatinine (mg/dl)	0.80 ± 0.2	0.82 ± 0.2	< 0.001	0.86 ± 0.3	< 0.001
eGFR (ml/min)	96.1 ± 20.7	93.0 ± 20.4	< 0.001	84.9 ± 21.0	< 0.001

Data are reported as means±SD. *N* number of individuals, *BMI* body mass index, *T2DM* type 2 diabetes, *eGFR* glomerular filtration rate, *LDL* low-density lipoprotein

### Renal Function Outcomes

#### Serum Creatinine, Glomerular Filtration Rate, and Albuminuria

S-creatinine increased at visit 1 (0.80 ± 0.2 versus 0.82 ± 0.2 mg/dL; *p < 0.001*) and at visit 2 (0.80 ± 0.2 versus 0.86 ± 0.3 mg/dL; *p < 0.001)* compared with baseline. Accordingly, eGFR declined at visit 1 (96.1 ± 20.7 to 93.0 ± 20.4 mL/min; *p < 0.001*) and visit 2 (84.9 ± 21.0 mL/min; *p < 0.001*). The mean postoperative change in eGFR was 3.1 ± 17.7 ml/min (*n.s*.) at visit 1 and 11.1 ± 15.9 ml/min (*p < 0.001)* at visit 2. Available data (*n* = 30) showed a prevalence of albuminuria of 27.0% at baseline. During follow-up, the prevalence initially remained stable at visit 1 and then decreased by trend to 20.0% (*n.s.)*.

### Subgroup Analysis

#### Baseline Classification and Postoperative Course of eGFR Levels Within each Subgroup

##### Reduced Filtration Rate

23.5% of patients had a reduced filtration rate, which was 72.5 ± 21.9 ml/min (mean ± SD) at baseline (presumably CKD stage 2). Patients were 43.1 ± 11.7 years (mean ± SD), S-creatinine was 1.0 ± 0.2 mg/dl (mean ± SD), 79% were suffering from treatment requiring hypertension, and 29% from T2D (mean HbA1c 6.1 ± 1.0%). eGFR initially increased to a mean eGFR of 80.2 ± 21.9 ml/min at visit 1 (*p < 0.05*) and thereafter remained stable with 69.1 ± 19.3 ml/min at visit 2 compared with baseline (*n.s.*), respectively.

##### Normal Filtration Rate

At baseline, normal filtration rate with a mean eGFR of 99.7 ± 13.3 ml/min was present in 60.7% of patients. Patients were 44.3 ± 10.4 years (mean ± SD), S-creatinine was 0.8 ± 0.8 mg/dl (mean ± SD), 63% were suffering from treatment requiring hypertension, 35% from T2D (mean HbA1c 6.2 ± 1.1%). eGFR remained stable with a mean eGFR of 97.1 ± 18.3 ml/min at visit 1 (*n.s.*) and thereafter significantly declined to 88.7 ± 19.4 ml/min at visit 2 (*p < 0.001*) compared to baseline, respectively.

##### Increased Filtration Rate

**Fig. 1:**
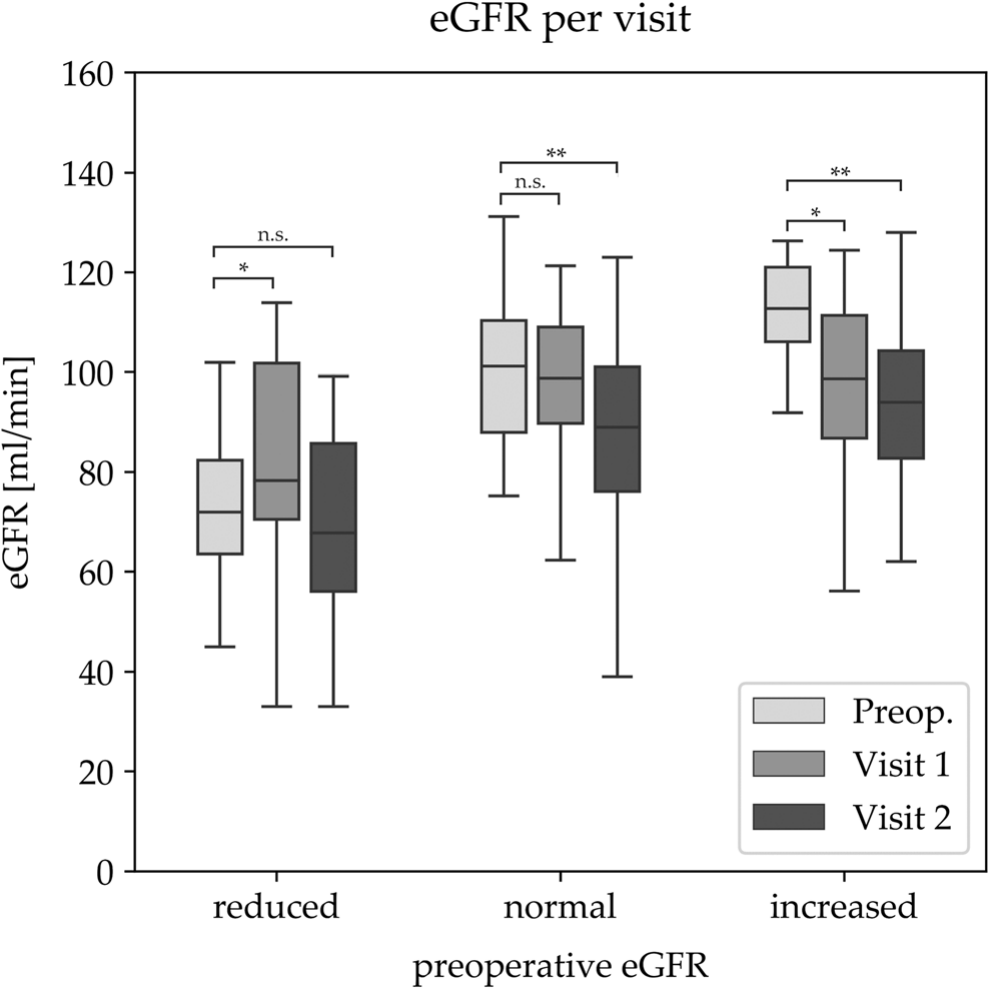
Subgroup classification according to eGFR at baseline and postoperative course of eGFR levels for each subgroup. *eGFR*, estimated glomerular filtration rate. *Preop.*, preoperative. * *p < 0.05*, ** *p < 0.001*

At baseline, increased filtration rate with a mean eGFR of 117.3 ± 20.7 ml/min was present in 15.7% of patients. Patients were 45.3 ± 11.6 years (mean ± SD), S-creatinine was 0.6 ± 0.1 mg/dl (mean ± SD), 63% were suffering from treatment requiring hypertension, 38% from T2D (mean HbA1c 5.9 ± 0.6%). Increased filtration rates decreased over time to 96.6 ± 19.4 ml/min at visit 1 (*p < 0.05*) and 94.2 ± 17.7 ml/min at visit 2 (*p < 0.001*) compared to baseline, respectively. Figure 1 shows the subgroup classification according to eGFR at baseline and the postoperative course of eGFR levels for each subgroup.

#### Adjustment for Age- and Sex-Related Decline in eGFR

**Fig. 2:**
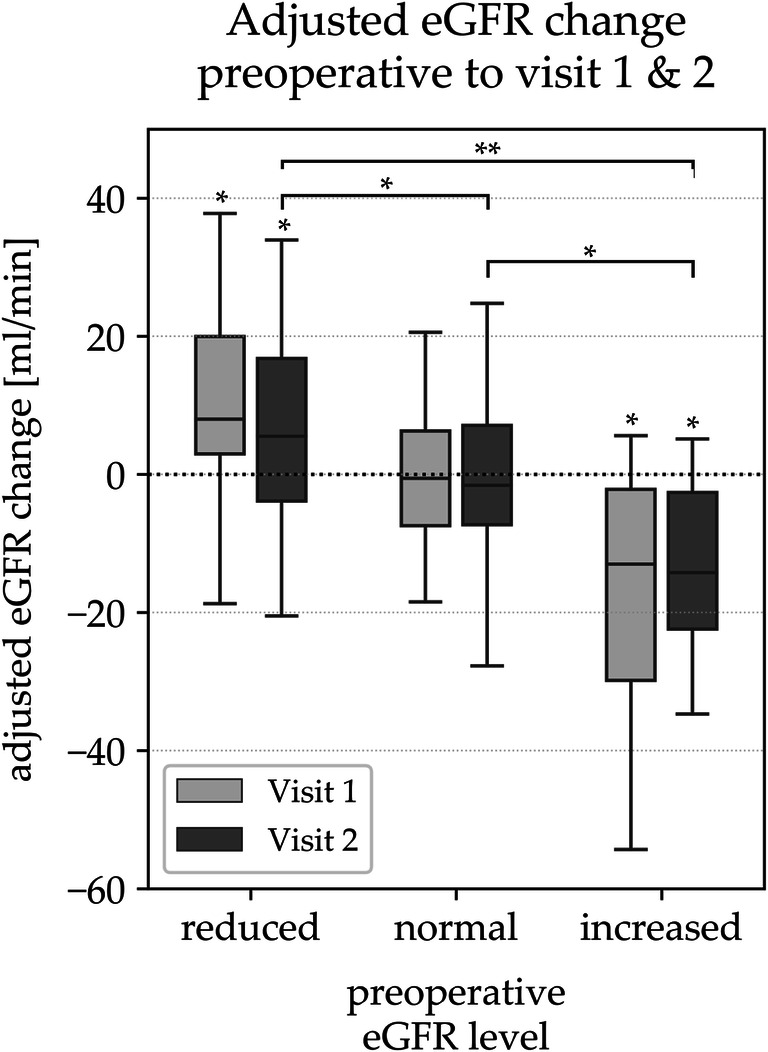
shows the subgroup classification according to eGFR at baseline and the postoperative course of eGFR levels for each subgroup as well as comparison of change in eGFR between the subgroups at long-time follow-up after adjusting for age-and sex-related decline in eGFR. *eGFR*, estimated glomerular filtration rate. * *p < 0.05*, ** *p < 0.001*

After adjustments for age- and gender-related decline, eGFR significantly increased in the short-and long-term (9.3 ± 17.1 ml/min and 6.4 ± 14.4 ml/min, respectively; *p < 0.05*) among patients with reduced filtration rate. Among patients with normal filtration rate, eGFR remained stable during follow-up (−1.0 ± 12.6 ml/min at visit 1 and − 1.3 ± 15.2 ml/min and visit 2, *n.s.).* Among patients with increased filtration rate, eGFR significantly decreased in the short-term (−18.9 ± 22.5 ml/min; *p < 0.05)* and long-term (−13.2 ± 12.2 ml/min; *p = 0.001)* and remained within the normal range 8.2 years after surgery (Fig. 2).

##### Comparison of Change in Adjusted eGFR Between the Subgroups at Long-Time Follow-Up

The level of adjusted eGFR and its magnitude of change over long-time follow-up were compared between the subgroups (Fig. 2). Patients with a reduced filtration rate showed a significant increase in adjusted eGFR compared with patients with a normal filtration rate (*p < 0.05*), in whom adjusted eGFR remained stable, and compared with patients with an increased filtration rate (*p* < 0.001). Patients with an increased filtration rate showed a significant decrease in adjusted eGFR compared with patients with a normal filtration rate (*p < 0.05*).

### Univariate Regression Analysis for Change in Adjusted eGFR

**Fig. 3:**
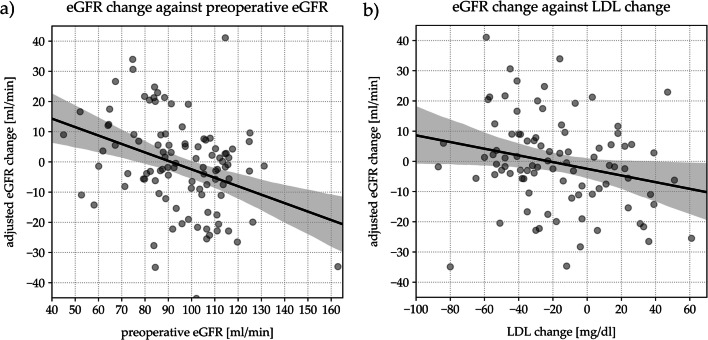
Univariate regression analysis between change in adjusted eGFR at long-time follow-up and the following variables: (**a**) preoperative eGFR and (**b**) LDL-cholesterol change. e*GFR* estimated glomerular filtration rate, *LDL* low-density lipoprotein

After adjustment for age- and gender-related decline in eGFR, change in eGFR at long-time follow-up showed a significant negative correlation with eGFR at baseline (B = −0.28; *p < 0.001*) and change in LDL-cholesterol over time (B = -0.11; *p < 0.05*). The lower the level of eGFR at baseline, the greater the increase in eGFR at long-time follow-up. The greater the decrease in LDL-cholesterol, the lower the decline in eGFR at long-time-follow up. Results of univariate regression analysis are graphically shown in Fig. 3.

### Multivariate Analysis: Predictors of Adjusted eGFR Change at Follow-Up

Univariate and multivariate analyses showed that preoperative eGFR, change in LDL-cholesterol, and hypertension requiring treatment were the only variables significantly associated with the change in adjusted eGFR (Table 2). Change in adjusted eGFR at long-time follow-up showed a significant negative correlation with eGFR at baseline (*B* = −0.31; *p < 0.001*), change in LDL-cholesterol over time (*B* = −0.09; *p < 0.05*), and a negative correlation with treatment requiring hypertension (B = −9.36; *p = 0.001*).

**Table 2:** Univariate and multivariate regression analyses to identify the factors determining the change in adjusted eGFR at long-time follow-up

**Univariate Analysis**	B	*p* value	Lower Bound	Upper Bound	R^2^	Adjusted R^2^
Age at baseline	−0.46	0.75	−0.33	0.24	0.001	
BMI at baseline	−0.10	0.54	−0.43	0.23	0.004	
BMI change	−0.05	0.82	−0.49	0.39	0.001	
HbA1c at baseline	−0.16	0.92	−3.23	2.95	0	
HbA1c change	2.86	0.15	−1.03	6.74	0.024	
eGFR at baseline	−0.28	<0.001	−0.42	−0.14	0.137	
LDL-cholesterol at baseline	0.03	0.56	−0.07	0.13	0.004	
LDL-cholesterol change	−0.11	<0.05	−0.21	−0.02	0.056	
Treatment requiring hypertension	−5.92	0.06	−12.05	0.21	0.035	
Diabetes at baseline	−5.86	0.07	−12.27	0.55	0.032	
**Multivariate model**					0.31	0.28
Constant	33.04	<0.001	19.57	46.52		
eGFR at baseline	−0.31	<0.001	−0.44	−0.19		
LDL-cholesterol change	−0.09	<0.05	−0.18	−0.10		
Treatment requiring hypertension	−9.36	0.001	−14.86	−3.871		

*B* correlation coefficent, *R*^*2*^ R squared, *BMI* body mass index, *eGFR* glomerular filtration rate, *LDL* low-density lipoprotein

## Discussion

In short- and mid-term studies (observation period of up to 4 years), improvement of eGFR profile has almost consistently been observed in patients with and without chronic renal failure following BS [7]. These beneficial renal effects were accompanied by relevant improvements in blood pressure and glycemic control that persisted for 1–5 years after surgery [3]. This is of interest as our long-term results (up to 11 years) are broadly in line with the short- and mid-term trends observed.

In our study population, eGFR declined by 11.1 ml/min over a median follow-up of 8.5 years, which translates into an eGFR decline of 1.31 ml/min per year. Since with advancing age per se renal function declines physiologically, the observed eGFR decline does resemble the physiological loss of functioning nephrons [19, 20].

There was a negative correlation between eGFR change at long-time follow-up and preoperative eGFR as seen in previous short-and mid-term studies [10, 13]. According to our results, patients with reduced glomerular filtration seemed to benefit most from the renoprotective effect of bariatric surgery, which is reflected by a significant increase of eGFR at long-time follow-up after adjusting for age- and gender-related decline in eGFR. A 5-year prospective cohort study reported similar findings stating that the subgroup of patients with established renal impairment showed an increase in eGFR overall and seemed to have the greatest benefit from BS in terms of renoprotection. In those with normal renal function, eGFR maintenance could be achieved for at least 5 years after RYGB [21]. Similarly, we found that in severely obese patients with normal glomerular filtration rate at baseline, bariatric surgery stabilized renal function in the long term after adjusting for age-and gender-related decline in eGFR. Furthermore, patients with increased filtration rate at baseline showed a decline of eGFR to normal levels at long-time follow-up. In this subgroup, BS resulted in an improvement of renal function preventing from progressive eGFR decline as usually seen in obesity-related hyperfiltration. Similar results were found in a multiethnic Asian population, where reversal of both, hypo -and hyperfiltration, could be achieved 1 year after BS [22]. As discussed in a systematic review by Bilha et al., the beneficial effects of BS in patients with hypofiltration are of particular interest [7]. In our cohort, 79% of patients with reduced filtration rate were suffering from treatment requiring hypertension and almost a third from T2D at baseline. We can only speculate that the resolution of these comorbidities as a result of weight loss is the main driver for renal function improvement in this subgroup at increased cardiovascular risk.

We did not measure cystatin C or creatinine clearance, which are marker of renal function less sensitive to changes in muscle mass than S-creatinine. Even though declines in lean mass can be observed after bariatric surgery in men and women, these changes in body composition occur mainly during the early postoperative phase and will already be followed by an increase in muscle strength 6 and 12 months postoperatively [23].

In the CREDENCE trial, eGFR slope per year was −4.6 ml/min in patients with T2D and kidney disease, while treatment with the SGLT-2 inhibitor canagliflozin led to an eGFR slope reduction by 2.7 ml/min per year (eGFR slope − 1.85 ml/min per year) [24]. Thus, the renoprotective effect of SGLT-2 inhibitors slowed progression to ESRD by approximately 11.6 years. Over a median follow-up of 8.5 years, we assumed a minimum gain of time to ESRD of 5.4 years in patients with reduced filtration rate adjusted for the observed eGFR decline of 3.4 ml/min between baseline and long-time follow-up. If using a less conservative assumption, we can only speculate, that BS may be able to exert similar renoprotective effects in our cohort in the long-term as treatment with SGLT-2 inhibitors.

We can also report that the decline in LDL-cholesterol following BS showed a negative correlation with adjusted eGFR change at long-time follow-up. Our results are supported by the evidence that human mesangial cells exposed to LDL-cholesterol dramatically increase synthesis of mesangial matrix components, so that glomerular basement membrane thickness directly correlates with circulating levels of cholesterol [25]. Thickening of glomerular basement membrane is considered a hallmark of e.g. diabetic nephropathy and leads to decline in renal function [26]. Moreover, lipids also directly damage podocytes [25, 27]. Podocyte failure results in adaptive focal segmental glomerulosclerosis, hyperfiltration, and proteinuria. No clinical studies have explicitly addressed the role of ectopic lipid accumulation in ORG. However, interventions targeting cellular lipid metabolism have been shown to reduce glomerular injury in experimental models by reducing ectopic lipid accumulation. Statin studies in CKD have shown mixed results, but these did not specifically address ORG [27]. Since it can be speculated that the majority of our patients can be classified as being high risk patients with a LDL-cholesterol treatment goal < 70 mg/dl according to the current guidelines for the management of dyslipidemias [28], our findings about the negative association between decline in LDL-cholesterol and eGFR change are of particular clinical interest. Our data suggest that in terms of renoprotection, even less aggressive LDL-cholesterol lowering < 100 mg/dl may be clinically relevant in the long term.

Patients with treatment-requiring hypertension showed a worse adjusted eGFR outcome. Accordingly, in patients with biopsy-proven mild ORG and normal renal function, improvement of renal function variables seemed to be driven by an improvement of hypertension following surgery regardless of the type and the number of glomerular lesions [9]. In contrast, Schuster et al. stated that the presence of hypertension did not reduce the likelihood of improvement of renal function measured by S-creatinine. However, it remained to be determined whether the absence of hypertension would have allowed for a greater extent of improvement in S-creatinine [29].

Our study has a few limitations. First, our study is a retrospective cohort study that lacks a control group. Since data have not been collected prospectively, internal and external validity may be impaired. It must also be noted that the reported eGFR is the best estimate of a patient’s GFR, but not the patient’s actual GFR, which could have impacted the results.

## Conclusion

Our results underline the relevance of renal impairment in patient selection for BS, since BS may contribute to effective renoprotection in the long-term.

## Abbreviations

BMI: Body mass index
BS: Bariatric surgery
CKD: Chronic kidney disease
CKD-EPI: Chronic kidney disease epidemiology collaboration
eGFR: Estimated glomerular filtration rate
ESRD: End stage renal disease
HbA1c: Hemoglobin A1c
LDL: Low-density lipoprotein
ORG: Obesity-related glomerulopathy
Preop.: Preoperative
RYGB: Roux-en-Y gastric bypass
SD: Standard deviation
SG: Sleeve gastrectomy
SGLT-2: Sodium/glucose cotransporter 2
T2D: Type 2 Diabetes mellitus
